# Optic Nerve Schwannoma: A Report of a Rare Case From India and Literature Review

**DOI:** 10.7759/cureus.59824

**Published:** 2024-05-07

**Authors:** Abhay Lune, Renu Magdum, Supriya Pokle, Megha R Kotecha, Vishakha Vatkar

**Affiliations:** 1 Ophthalmology, Dr. D. Y. Patil Medical College, Hospital & Research Centre, Pune, IND

**Keywords:** india, case report, orbital tumors, optic nerve sheath, schwannoma

## Abstract

Optic nerve schwannoma is a very rarely occurring tumor described in the literature. It is due to the fact that the optic nerve is myelinated by oligodendrocytes. Schwannomas are tumors of the peripheral nervous system, hence optic nerve schwannoma is a rare phenomenon. A 34-year-old patient presented in the outpatient department with complaints of gradual painless protrusion of the left eye (LE) for the past one year. There was no history of diminution of vision. On examination, vision in both eyes was 6/6, anterior segment examination in both eyes was normal, and pupils were central, circular, and reacting to light. Intraocular pressure was measured on a noncontact tonometer and was within normal range. Both eyes' optic disc, fundus, and visual fields were normal. On inspection, axial proptosis was noted in the LE. Proptosis measurement (on Hertel exophthalmometer) in the right eye was 17 mm and in the left eye was 21 mm.

MRI of the orbit without contrast was done and showed a well-defined, soft tissue lesion of the optic nerve in the intraconal compartment of the left orbit. Surgical excision of the tumor was done by lateral orbitotomy approach and the tumor was removed in total. Histopathological examination of the mass revealed a benign spindle cell neoplasm suggestive of schwannoma.

Postoperatively, proptosis was resolved, 17 mm both in the right and left eye (on Hertel exophthalmometer), and vision in LE remained unchanged (6/6). Postoperatively, intraocular pressure (on noncontact tonometer) was within normal range, and the optic disc, fundus, and visual fields were normal.

## Introduction

Schwannomas are typically benign growths that are well-defined and commonly connected to peripheral nerves. They are composed of a group of Schwann cells, often experiencing cystic and degenerative alterations. Schwannomas are typically found as single tumors and frequently impact the smaller peripheral nerves located in the head, neck, and inner surfaces of the limbs [1]. Although it constitutes 8% of all intracranial tumors, schwannoma of the optic nerve is a rare phenomenon and limited reports are available in the literature [2-4]. In terms of clinical presentation, these tumors may manifest with gradual proptosis, and in rare instances, they can lead to visual field impairment or even blindness. Additionally, they may be accompanied by symptoms such as headaches and discomfort around the retro-orbital area.

## Case presentation

**Figure 1:**
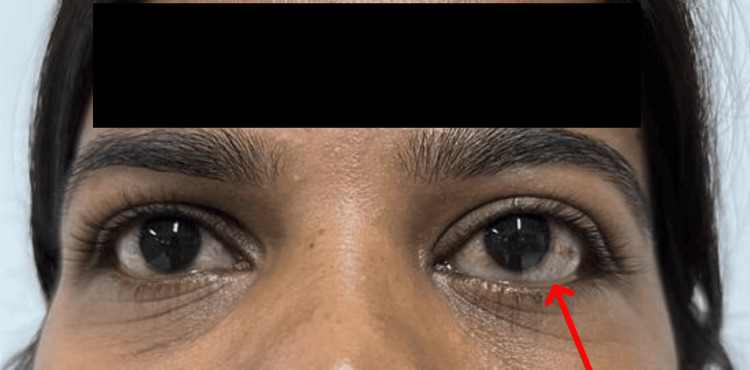
Left eye axial proptosis was noted. On inspection, axial proptosis was noted in the left eye.

A 34-year-old female patient came to the outpatient department (OPD) with complaints of gradual painless protrusion of the left eye (LE) for one year. There was no history of diminution of vision, double vision, any history of ocular trauma, or any previous ocular surgery. No history of thyroid disease or any other systemic illness was reported. No family history of neurofibromatosis was present. Systemic examination was normal and no signs of neurofibromatosis were seen. Vision in both eyes was 6/6 on Snellen’s chart, and color vision was intact in both eyes. Extraocular muscle movements were free, full, and painless. Intraocular pressure measured on the noncontact tonometer was 12 mmHg in the right eye and 14 mmHg in the left eye. On inspection, axial proptosis was noted in the LE (Figure 1). Lagophthalmos and lid lag on the downgaze were absent. There was no increase of proptosis on bending or with the Valsalva maneuver. On palpation, the local rise of temperature, local tenderness, pulsations, and thrill were absent. Orbital margins were intact and no resistance to retropulsion was noted. No enlargement of preauricular or submandibular lymph nodes was found. Auscultation found no bruits. Hertel exophthalmometer confirmed a measurement of 17 mm in the RE and 21 mm in the LE, so LE proptosis was confirmed. Anterior segment evaluation on the slit lamp was normal and pupils were central, 3 mm in size, circular, and reacting to light in both eyes. Both eyes' optic disc, fundus, and visual fields were within normal limits. All routine blood investigations and thyroid function tests were done, which were within normal limits.

**Figure 2:**
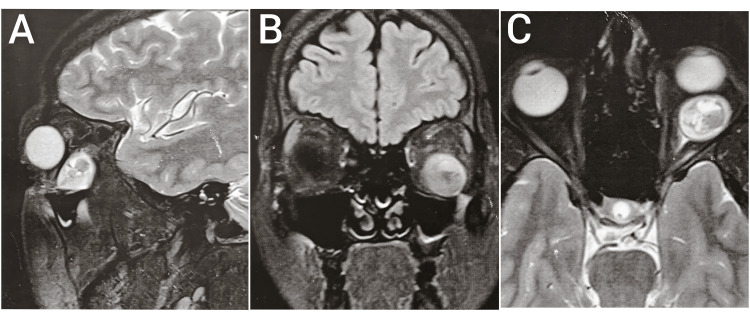
MRI of the orbit without contrast. Figures A (T2-weighted), B (fluid-attenuated inversion recovery), and C (T2-weighted) denoting sagittal, coronal, and axial sections of MRI of the orbit without contrast, respectively, showing a well-defined, oval-shaped, soft tissue lesion in the intraconal compartment in the left orbit.

MRI of the orbit without contrast was done and revealed a well-defined, oval-shaped, soft tissue lesion in the intraconal compartment of the left orbit, measuring 1.9 x 2.1 x 1.6 cm, causing proptosis and mass effect over the adjacent left inferior and lateral rectus muscle. It was displacing the inferior rectus muscle medially and displacing the left eyeball anteriorly. It was causing compression of the intraconal portion of the left optic nerve (Figure 2). The above findings were suggestive of an optic nerve tumor.

**Figure 3:**
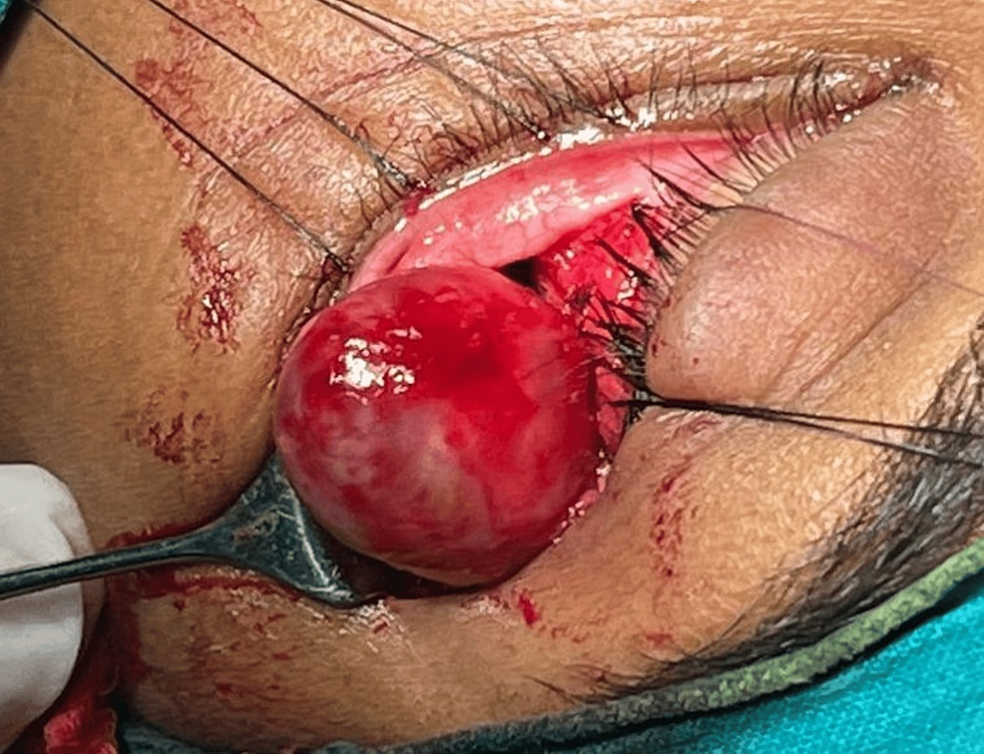
Intraoperative photograph while removing the mass present in the left orbit. The mass present in the intraconal compartment in the left orbit was removed surgically by the lateral orbitotomy approach.

Surgical excision and biopsy of the tumor were done by lateral orbitotomy approach (Figure 3). The incision site was marked and the incision was done at the lateral canthus with a 15-number blade, and lateral canthotomy was done. Dissection was done through the periorbita and then the intermuscular septum; retrobulbar space was accessed. The tumor was present around the optic nerve and was removed in total.

**Figure 4:**
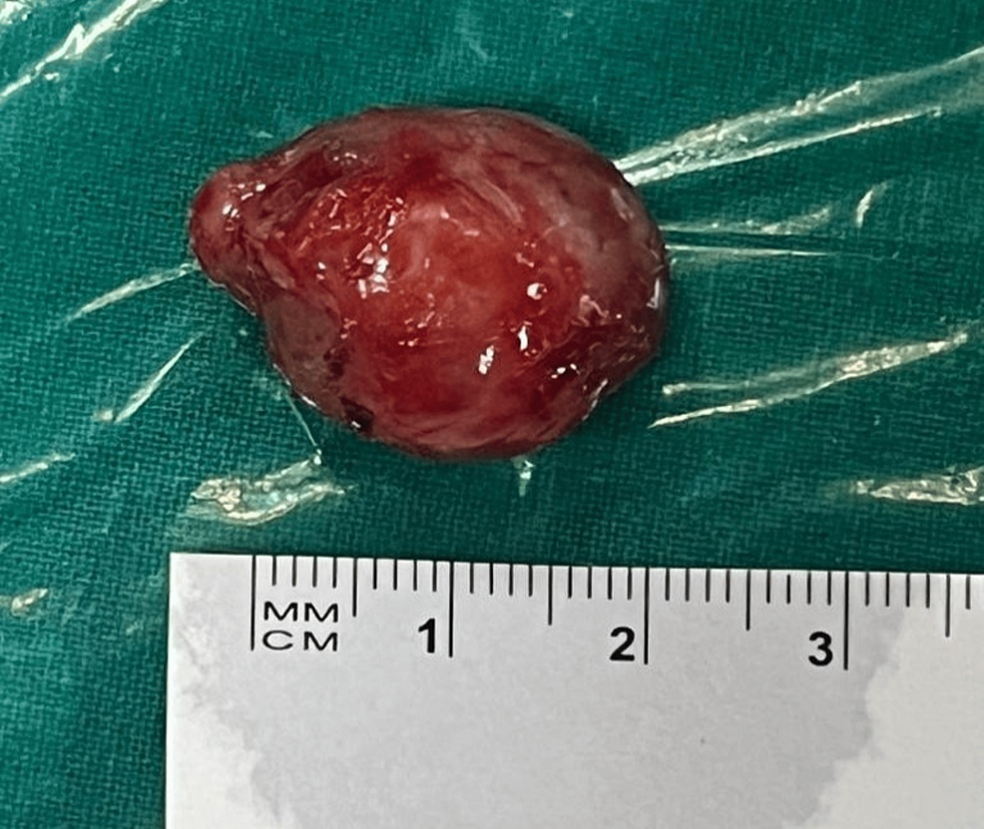
A single, greyish-white nodular mass is seen measuring approximately 1.9 x 2 x 1.7 cm. A single, greyish-white nodular mass measuring approximately 1.9 x 2 x 1.7 cm present in the intraconal compartment of the left orbit was removed by lateral orbitotomy approach.

**Figure 5:**
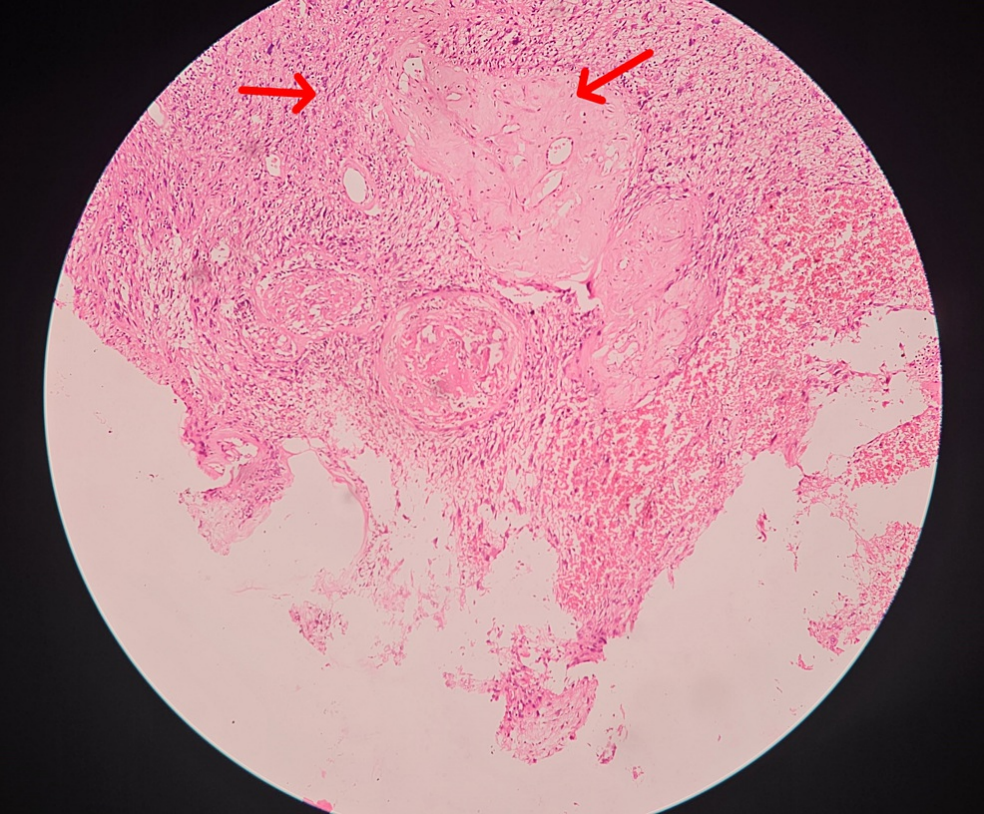
Histopathological examination slide (HPE) of the mass showing solid area (hypercellular area) and myxoid area (hypocellular area). Histopathological examination slide (HPE) of the mass showing the solid area (hypercellular, showing Antoni A) and myxoid area (hypocellular, showing Antoni B). The hypercellular area shows spindle cells arranged in an interlacing pattern.

**Figure 6:**
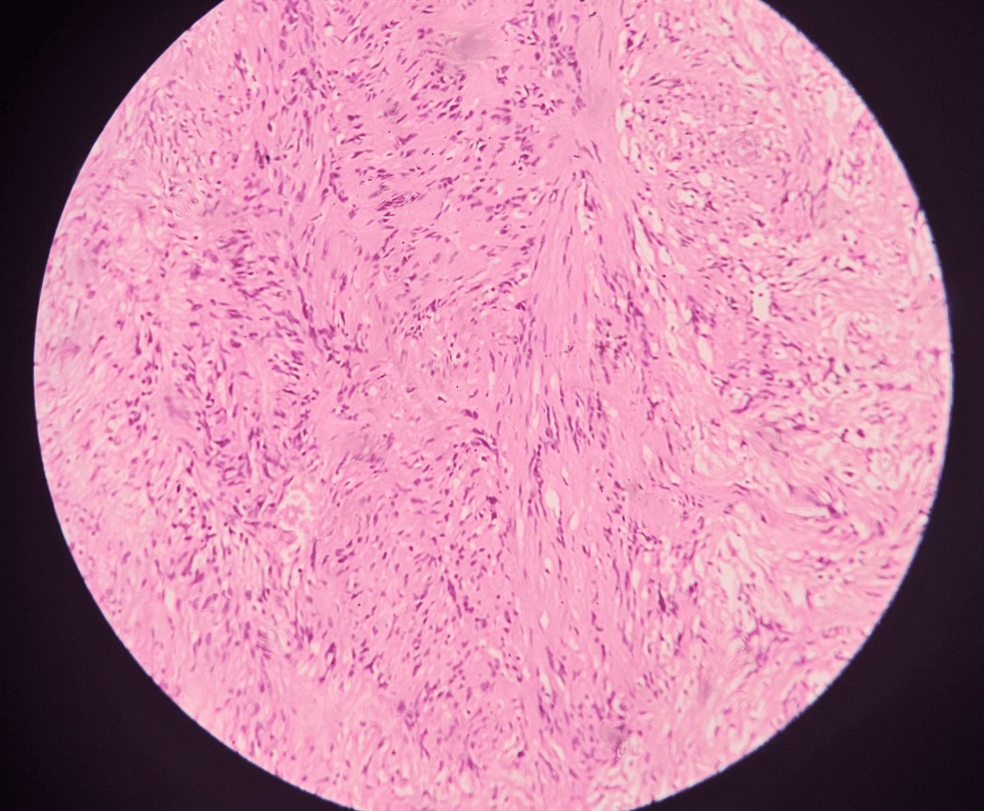
Histopathological examination slide (HPE) of the mass with high magnification of hypercellular area. Histopathological examination slide (HPE) of the mass shows the hypercellular area with spindle cells arranged in interlacing fascicles and bundles.

**Figure 7:**
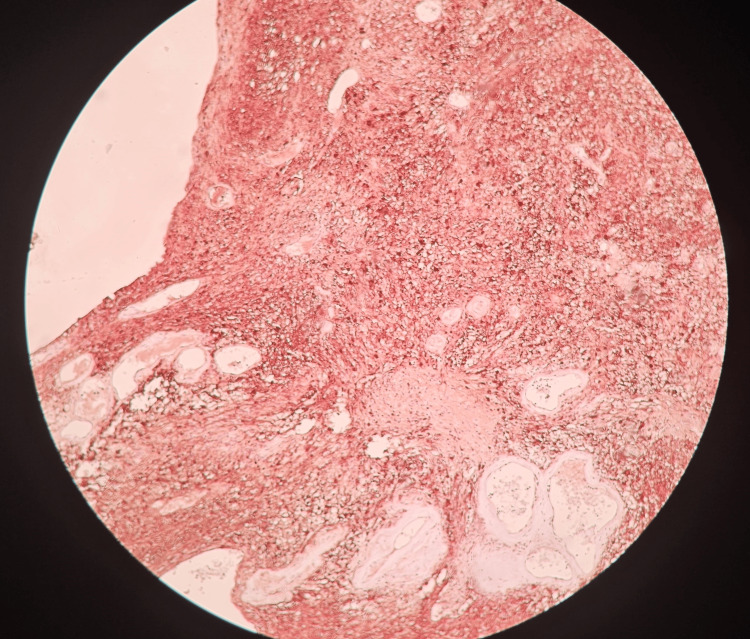
Histopathological slide of the mass stained with S-100 stain. Positive results are indicative of a peripheral nerve sheath tumor.

**Figure 8:**
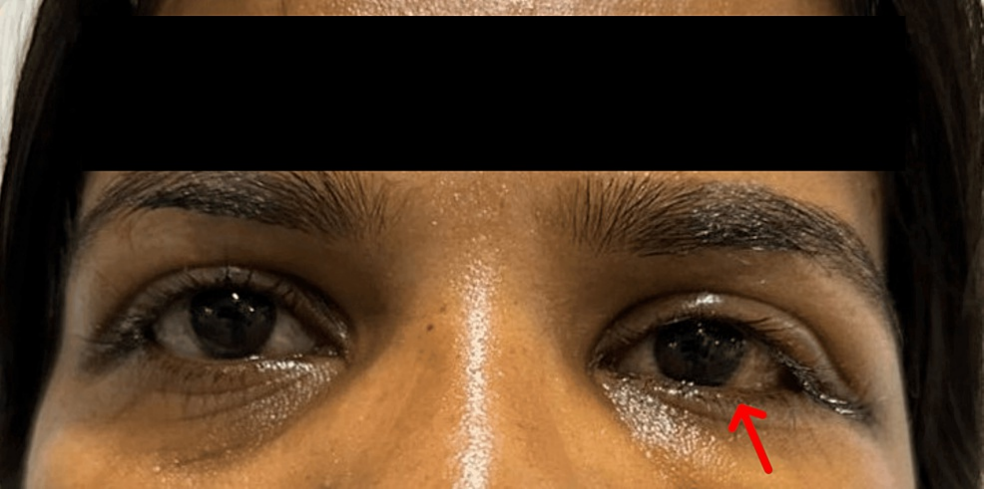
Image of the patient on postoperative day three following the left eye lateral orbitotomy procedure. Left eye proptosis was resolved following surgery (image of the patient on postoperative day three).

On gross examination, a 1.9 x 2 x 1.7 cm single, grey-white nodular mass was present (Figure 4). Histopathological examination (HPE) of the mass showed an encapsulated tumor comprising hypo and hypercellular areas. Hypercellular areas reveal spindle cells arranged in interlacing fascicles and bundles with focal palisading noted. Individual tumor cells revealed elongated nuclear tapering ends. There was no evidence of increased or atypical mitosis or necrosis in the sections studied showing a solid area (hypercellular) showing Antoni A, and a myxoid area (hypocellular) showing Antoni B (Figures 5, 6). S100 staining was positive. S-100 protein is a marker for peripheral nerve sheath tumors and it is positive in all benign Schwann cell tumors (Figure 7). All these features were suggestive of benign spindle cell neoplasm suggestive of schwannoma. Following the procedure, proptosis was resolved completely with good cosmetic results and vision in the LE remained unchanged (6/6), pupils were central, 3-4 mm in size, circular, and reacting to light (Figure 8). Postoperative optic disc, fundus, and visual fields were normal, and postoperative intraocular pressure was 14 mmHg in the right eye and 16 mmHg in the left eye (on a noncontact tonometer).

## Discussion

Optic nerve schwannoma is a rare tumor that arises from hyperplasia of the myelin-producing Schwann cells. It typically functions to provide myelination to peripheral neurons starting at the Obersteiner-Redlich zone, which marks the transition from the central to the peripheral nervous system [5]. The rarity of the tumor in the optic nerve can be attributed to the lack of Schwann cells in the optic nerve.

Optic nerve schwannoma is believed to potentially originate from Schwann cells that have migrated away from their usual location in the neural crest, as well as Schwann cells that typically surround the sympathetic nerves that supply blood vessels within the orbit. The precise location of nerve origin may not be identifiable, even during surgical procedures [3]. Another, although less probable, alternative explanation is that the optic nerve may occasionally contain atypical Schwann cells that are susceptible to becoming cancerous. Nevertheless, reliable studies in the past have investigated the origin and myelination pattern along the optic nerve, suggesting that oligodendrocytes are indeed responsible for myelinating the optic nerves in a sequential manner from the proximal to the distal regions [6,7].

While neurofibromatosis (NF) type 2 is associated with schwannomas [8], the present case reported no such history or clinical features of NF. Painless protrusion of the affected eye (LE) was the only clinical feature present in our case, which was similar to the case reported by Sharma et al. [3].^ ^Blurred vision, reduced visual acuity, diplopia, distorted visual fields, and pain in the orbits have been reported in similar cases in the past [2,4,7].Young and middle-aged adults are the more commonly affected group, with studies reporting patients ranging from 29 to 48 years of age [2,3].Symptoms of rapid growth and discomfort can be indicators of cancerous development [2].

Optic nerve schwannomas are usually benign, unilateral, and localized [9].^ ^Schwannomas are usually seen in the middle age group with a median age group of 40 years [10]. Surgical excision offers a complete cure. Benzalim et al. undertook a partial excision owing to optic nerve adherence [2]. External beam radiotherapy was added along with orbital excision in one of the cases reported by Kashkouli et al. [4]. Incomplete surgical removal of the tumor often leads to its recurrence. Nonetheless, when the tumor extends to the orbital apex, it is advisable to leave the apical part untouched to prevent damage to the elements traversing the orbital apex and the superior orbital fissure. Schwannomas of the optic nerve that are tightly adherent to the nerve typically result in a negative prognosis for the vision [4]. While our patient's vision remained unchanged following the surgical procedure, Benzalim et al. reported no improvement although a resolution of proptosis was reported by them [2]. Kashkouli et al. reported no improvement in visual acuity in the long-term follow-up [4], while Ramey et al. reported a recurrence of visual deficits following the procedure [7].

## Conclusions

We reported a rare case of optic nerve schwannoma in a middle-aged female from India, with positive outcomes showing resolution of the proptosis following the surgical excision. However, long-term follow-up of the patient needs to be undertaken to understand the prognosis of the condition and rule out chances of recurrence.
